# Identifying and supporting students at risk of failing the National Medical Licensure Examination in Japan using a predictive pass rate

**DOI:** 10.1186/s12909-020-02350-8

**Published:** 2020-11-10

**Authors:** Koji Tsunekawa, Yasuyuki Suzuki, Toshiki Shioiri

**Affiliations:** 1 Department of Institutional Research for Medical Education, Gifu University Graduate School of Medicine, Yanagito 1-1, Gifu City, 501-1194 Japan; 2 Medical Educational Development Center, Gifu University, Gifu, Japan; 3 Department of Psychiatry and Psychotherapy, Gifu University Graduate School of Medicine, Gifu, Japan

**Keywords:** National Medical Licensure Examination, Logistic regression analysis, Predicting student failure, Supporting high-risk students

## Abstract

**Background:**

Students who fail to pass the National Medical Licensure Examination (NMLE) pose a huge problem from the educational standpoint of healthcare professionals. In the present study, we developed a formula of predictive pass rate (PPR)” which reliably predicts medical students who will fail the NMLE in Japan, and provides an adequate academic support for them.

**Methods:**

Six consecutive cohorts of 531 medical students between 2012 and 2017, Gifu University Graduate School of Medicine, were investigated. Using 7 variables before the admission to medical school and 10 variables after admission, we developed a prediction formula to obtain the PPR for the NMLE using logistic regression analysis. In a new cohort of 106 medical students in 2018, we applied the formula for PPR to them to confirm the capability of the PPR and predicted students who will have a strong likelihood of failing the NMLE.

**Results:**

Medical students who passed the NMLE had the following characteristics: younger age at admission, graduates of high schools located in the surrounding area, high scores in the graduation examination and in the comprehensive computer-based test provided by the Common Achievement Test Organization in Japan. However, total score of examination in pre-clinical medical sciences and Pre-CC OSCE score in the 4th year were not correlated with the PPR. Ninety-one out of 531 students had a strong likelihood of failing the NMLE between 2012 and 2017 and 33 of these 91 students failed NMLE. Using the PPR, we predicted 12 out of 106 students will have a strong likelihood of failing the NMLE. Actually, five of these 12 students failed NMLE.

**Conclusions:**

The PPR can be used to predict medical students who have a higher probability of failing the NMLE. This prediction would enable focused support and guidance by faculty members. Prospective and longitudinal studies for larger and different cohorts would be necessary.

**Supplementary Information:**

**Supplementary information** accompanies this paper at 10.1186/s12909-020-02350-8.

## Background

**I**n many countries such as the US, Germany, and Japan, medical students need to pass the National Medical Licensure Examination (NMLE) in order to take a physician’s license, and students who fail to pass the NMLE pose a huge problem from the educational standpoint of healthcare professionals [1–6]. Students failing USMLE Step 1 are often delayed from continuing course work, which affects their graduation and increases costs [4]. Failing Step 1 can also affect a student’s ability to enter a residency program and in some instances restrict them from applying for residency in specific states [4, 7]. Moreover, even if they enter a residency program, their performance in several specialty board examinations was poorer than those who passed the USMLE or the National Board of Medical Examiners (NBME) without failing [8–12]. There is also a concern about repeaters who cannot pass the NMLE and repeatedly take the examination. The pass rate among such repeaters is not sufficient, while the pass rate among those who take the examination the first time is very high: 67% vs 96% in USMLE Step 1 in 2017, and 63.9% vs 93.3% in the NMLE in Japan in 2018 [13, 14]. Those who fail seem to be much more likely to end up as repeaters.

Most studies in the past decade have primarily focused on the outcome [3, 5] or poor performance [2, 6] of those who fail USMLE or NMLE. To the best of our knowledge, there are only two studies in the US and Netherlands that have attempted to create models for predicting those who will fail among first-time test takers of Step 1 [4], and in the first-year undergraduate medical curriculum [1]. However, these studies did not confirm their results using predictors in a new group of students. Previous studies about medical students’ academic success reported academic performance associated with not only post admission variables such as previous academic performance [15, 16] and Objective Structured Clinical Examination (OSCE) [17], but also pre-admission variables such as gender [17], age at admission [16], hometown [16, 18], type of high school (HS) [19], HS grade point average (GPA) [20], and entrance exam for medical schools [21].

Therefore, the goal of the current study was to develop a model that can reliably predict those who would fail the NMLE using a prediction formula for the pass rate of NMLE: predictive pass rate (PPR). The prediction formula was then applied to a new cohort of medical students to identify students who had a high risk and need support.

### Current situation of medical education and the number of doctors in Japan

Undergraduate medical education in Japanese medical schools is usually 6 years [22, 23], including 4 years of pre-clinical medical sciences and 2 years of clinical training. Graduates from these medical schools can take the NMLE.

In Japan, the total number of enrolling medical students has been controlled by government every year. As a result, entering medical school is highly competitive, and almost all students who pass the entrance examination potentially have the academic ability to pass NMLE. Furthermore, the number of doctors in Japan also has been controlled by the government due to the assumption of the number of doctors in future demand the future. The number of medical students per year has been kept around 7600 in the past two decades (1990th and 2000th). However, according to Organization for Economic Co-operation and Development (OECD) data, Japan is ranked 28th in terms of the number of practicing doctors among 35 OECD countries [24], and the number of medical students was also the least among OECD countries [25]. Since 2008, the number of medical students has been gradually increased to about 9400. However, even this effort is not enough especially in rural areas. Therefore, increasing the number of NMLE failures at rural universities will add to the shortage of physicians and the uneven regional distribution of physicians, which will ultimately affect Japan’s healthcare system.

## Methods

### Participants

To develop a reliable PPR for the NMLE, six consecutive cohorts of 531 students (2012–2017, 6th academic year) of the Gifu University School of Medicine (GUSM) were included. The GUSM is one of 51 public schools that are largely supported by the Japanese government. The cohorts in each academic year comprised 78, 69, 84, 97, 110, and 93 students, respectively. Through a prospective study, a cohort of 106 students in 2018 was investigated to confirm the PPR by predicting which students will have a strong likelihood of failing the NMLE and providing remediation to them (Table 1). Data from the 637 students in 2012–2018 were obtained after the students were anonymized by the academic affairs office of the GUSM. Ethical approval was granted by the GUSM Ethics Committee. Anonymity and confidentiality were guaranteed (date: 11/30/2016, reference number: 28–333).

**Table 1:** Characteristics of students in the 2012–2017 and 2018 cohorts

Variables	2012–2017 (***n*** = 531)			2018 (***n*** = 106)	
	Failed (%)	Passed (%)	***P***-value	Failed (%)	Passed (%)
Gender					
Male	34 (8.4)	371 (91.6)	0.0071	4 (5.1)	75 (94.9)
Female	2 (1.6)	124 (98.4)		1 3.7)	26 (96.3)
Age at admission	27.11 ± 7.51	19.67 ± 3.65	< 0.0001	33.00 ± 10.61	19.16 ± 3.15
HS location			< 0.0001		
Neighborhood	5 (1.6)	307 (98.4)		1 (1.4)	69 (98.6)
Distant	31 (14.2)	188 (85.8)		4 (11.1)	32 (88.9)
Type of HS			0.3922		
Private	19 (7.8)	224 (92.2)		2 (4.4)	43 (95.6)
Public	17 (5.9)	271 (94.1)		3 (4.9)	58 (95.1)
Academic Level of HS	68.91 ± 5.88	69.22 ± 5.67	0.755	66.6 ± 7.77	68.66 ± 5.24
GPA in HS	4.15 ± 0.47	4.46 ± 0.45	< 0.0001	4.53 ± 0.11	4.50 ± 0.45
NCTUA score	83.97 ± 5.16	86.43 ± 3.94	0.00046	86.12 ± 2.11	86.64 ± 3.37
TOEFL score	515.0 ± 28.3	521.7 ± 25.0	0.124	538.8 ± 23.4	520.8 ± 25.1
Academic performance in liberal arts	68.81 ± 19.67	67.86 ± 13.44	0.695	79.40 ± 22.74	88.09 ± 19.44
Total score in basic sciences in the 1st year	73.70 ± 4.14	75.07 ± 4.62	0.085	74.29 ± 5.46	74.26 ± 4.76
Total score in basic biomedical sciences in the 2nd year	66.91 ± 4.45	72.75 ± 6.86	< 0.0001	64.43 ± 1.84	71.59 ± 6.61
Pre-clinical medical sciences in 3rd to 4th year	74.13 ± 4.71	75.99 ± 5.18	0.0370	72.73 ± 3.87	74.70 ± 6.48
CBT-IRT score in the 4th year	47.94 ± 8.98	59.53 ± 10.01	< 0.0001	49.74 ± 5.64	60.55 ± 10.92
Pre-CC OSCE score in the 4th year	4.26 ± 0.42	4.49 ± 0.38	0.00058	3.98 ± 0.37	4.53 ± 0.43
Performance in clinical clerkship in the 5th to 6th year	3.67 ± 0.72	4.01 ± 0.41	< 0.0001	4.43 ± 0.39	4.21 ± 0.52
Graduation examination in the 6th year	−1.33 ± 0.70	0.10 ± 0.95	< 0.0001	− 2.24 ± 0.52	0.11 ± 0.89
Holdover during the 1st to 6th year.			0.00018		
+	10 (23.8)	32 (76.2)		2 (13.3)	13 (86.7)
-	26 (5.3)	463 (94.7)		3 (3.3)	88 (96.7)

*CBT-IRT* Computer-Based Testing with Item Response Theory, *HS* High school, *NCTUA* National Center Test for University Admissions, *Pre-CC OSCE* Pre-Clinical Clerkship Objective Structured Clinical Examination, *TOEFL* Test of English as a Foreign Language. Level of HS shows an average of values that quantified the information on the difficulty of entrance examinations in each HS, that is, a higher level means a higher-difficulty entrance examination. The variables above the dotted line show factors before admission, while the ones below the line represent those after admission

### Variables

The dependent variable was “failing to pass the NMLE.” Data were obtained from the department of academic affairs of the GUSM. Explanatory variables included pre-admission variables such as gender, age at admission, location of HS (neighborhood prefectures including Gifu and Aichi from where about 60% of students enter the GUSM, and distant prefectures including Tokyo, Osaka and other prefectures), type of HS (public/private), academic level of HS (Table 1), HS GPA (5-grade evaluation), and achievement (percentage of correct answers) in the common entrance examination for university (National Center Test for University Admissions, NCTUA). Post-admission variables were Test of English as a Foreign Language (TOEFL) score, academic performance (percentage) in liberal arts, total score (percentage) in basic sciences in the first year, total score (percentage) in basic biomedical sciences in the second year, total score (percentage) in pre-clinical medical sciences in the third and fourth years, score in the nationwide Computer-Based Testing with Item Response Theory (CBT-IRT; which assess pre-clinical education in the fourth year), average score (six-point scale) in the Pre-Clinical Clerkship OSCE (Pre-CC OSCE) in the fourth year, achievement (standardized deviation values) in the graduation examination in the sixth year, performance in clinical clerkship during the fifth and sixth years, and with or without holdover from first to sixth years (Table 1). These data were obtained from the office of academic affairs of the GUSM, and the average value calculated from the data not including the missing value was substituted for the missing value.

### Data analysis

First, we used Fisher’s exact tests and independent t-tests to compare demographic data before and after attending university between those who failed and passed the NMLE in the 2012–2017 cohort. Second, for the 2012–2017 cohort, logistic regression predicting the likelihood of passing the NMLE was used to calculate ORs and 95% confidence intervals (95% CIs) after simultaneously controlling for potential confounders. Third, we created a prediction formula to obtain the PPR using logistic regression analysis with all possible models. In order to guarantee the generality, two models using forced entry and stepwise method were created,

To confirm the suitability of this formulas, we used these formulas for the new cohort in 2018 to identify students who had a lower PPR in NMLE (95% or less; strong likelihood to fail the NMLE). SPSS ver. 23.0 Japan for Windows (SPSS Inc., Chicago, IL, USA) was used to perform statistical test. Two-tailed *p*-values of < 0.05 were considered significant.

### Support to a cohort of graduates in 2018

Firstly, 9 months before the NMLE, we noticed these risk factors to all students. And then, 3 months before the NMLE, a group of students with a higher risk to fail were referred to the academic affairs committee. The committee members held an individual face-to-face interview, elaborately reviewed their motivation and preparedness for the NMLE, gave advice on how to study, determined their educational environment (i.e., location, period of time to study, support by family and/or classmates, economical problems), and encouraged and advised them repeatedly.

## Results

### Characteristics of those who failed and passed the NMLE in the 2012–2017 cohort

Table 1 shows some significant differences in the demographic data and achievements before and after attending university. In terms of pre-admission variables, those who failed the NMLE showed the following characteristics: predominantly male, older at admission, HS in distant prefectures, lower HS GPA, and lower NCTUA score. After attending university, they had significantly lower scores in basic and pre-clinical medical sciences, CBT-IRT, and Pre-CC OSCE and poor performance in clinical clerkship and were predisposed to repeat a year in medical school (Table 1).

### Logistic regression analysis

Table 2a and b show the results of logistic regression of variables that predict the likelihood of passing the NMLE. Both results show that medical students who passed the NMLE showed the following characteristics: younger age at admission, HS located in Gifu and Aichi Prefecture, higher scores in CBT-IRT and graduation examination but not the total score in pre-clinical medical sciences, and better performance in clinical clerkship.

**Table 2:** Logistic regression predicting the likelihood of passing the NMLE

Variables	***B***	S.E.	Wald chi-square	***P***-values	Odd ratio (OR)	95% CI
**A: forced entry method**						
Gender	1.008	0.974	1.071	0.301	2.739	0.406–18.473
**Age at admission**	**−0.133**	**0.055**	**5.853**	**0.0156**	**0.875**	**0.786–0.975**
**HS location**	**1.603**	**0.72**	**4.96**	**0.0259**	**4.966**	**1.212–20.348**
Type of HS	−0.77	0.591	1.699	0.192	0.463	0.145–1.474
Academic Level of HS	−0.033	0.051	0.437	0.508	0.967	0.876–1.068
HS GPA	0.266	0.494	0.29	0.59	1.305	0.496–3.434
NCTUA score	0.031	0.063	0.234	0.629	1.031	0.911–1.168
TOEFL score	0.005	0.011	0.184	0.668	1.005	0.984–1.026
Academic performance in liberal arts	−0.021	0.019	1.277	0.258	0.979	0.944–1.016
Basic sciences	0.037	0.084	0.197	0.657	1.038	0.880–1.224
Basic biomedical sciences	0.094	0.091	1.073	0.3	1.099	0.920–1.312
**Pre-clinical medical sciences**	**−0.359**	**0.111**	**10.522**	**0.00118**	**0.698**	**0.562–0.867**
**CBT-IRT**	**0.161**	**0.047**	**11.665**	**0.00064**	**1.175**	**1.071–1.288**
Pre-CC OSCE	−0.051	0.7	0.005	0.942	0.95	0.241–3.746
**Performance in clinical clerkship**	**1.424**	**0.541**	**6.933**	**0.00846**	**4.155**	**1.439–11.994**
**Graduation exam**	**1.728**	**0.475**	**13.21**	**0.00028**	**5.629**	**2.217–14.293**
Holdover	0.682	0.757	0.81	0.368	1.977	0.448–8.720
AUC	0.970					
**B: stepwise method**						
**Age at admission**	**−0.155**	**0.047**	**10.895**	**0.00096**	**0.856**	**0.781–0.939**
**HS location**	**1.576**	**0.672**	**5.509**	**0.0189**	**4.837**	**1.297–18.039**
**Pre-clinical medical sciences**	**−0.254**	**0.085**	**9.012**	**0.00268**	**0.775**	**0.657–0.915**
**CBT-IRT**	**0.167**	**0.04**	**17.553**	**0.00003**	**1.181**	**1.093–1.277**
**Performance in clinical clerkship**	**1.454**	**0.475**	**9.537**	**0.00222**	**4.279**	**1.686–10.861**
**Graduation exam**	**1.701**	**0.424**	**16.089**	**0.00006**	**5.478**	**2.386–12.577**
AUC	0.967					

*AUC* Area under the curve, *CBT-IRT* Computer-Based Testing with Item Response Theory, *HS* High school, *NCTUA* National Center Test for University Admissions, *Pre-CC OSCE* Pre-Clinical Clerkship Objective Structured Clinical Examination, *TOEFL* Test of English as a Foreign Language. Bold letters and digits indicate significance (*p* < 0.05). The variables above the dotted line show factors before admission, while those below the line were after admission

### PPR in the NMLE

Given that the PPR in the NMLE is p/100, the logistic regression formula was provided in Additional file 1 in the Supplementary Information section.

Ninety-one out of 531 students from 2012 to 2017 had a lower PPR for NMLE using the both methods, and actually 34 of 91 students failed the NMLE.

### Prediction for the graduates in the 2018 cohort and support

Using the above formulas, we predicted students who will likely fail the NMLE and guided them (Table 3a, b). Twelve out of 106 students in 2018 were predicted as having lower PPR (Table 3a), and they were supported by the faculty members. Eleven students predicted as having lower PPR by the stepwise method (Table 3b**)** were all included in the 12 students predicted in Table 3a. Seven of 12 students passed the NMLE after obtaining support, and five students failed as predicted. Thus the pass rate of NMLE in 2018 was 95.3% (101/106) (national average: 90.1%). As compared to the pass rate of 88.2% in 2017 (82/93 students) (national average: 88.7%), better outcome was obtained. In both models, we could predict all five students who would fail; these were included among the high-risk students.

**Table 3:**
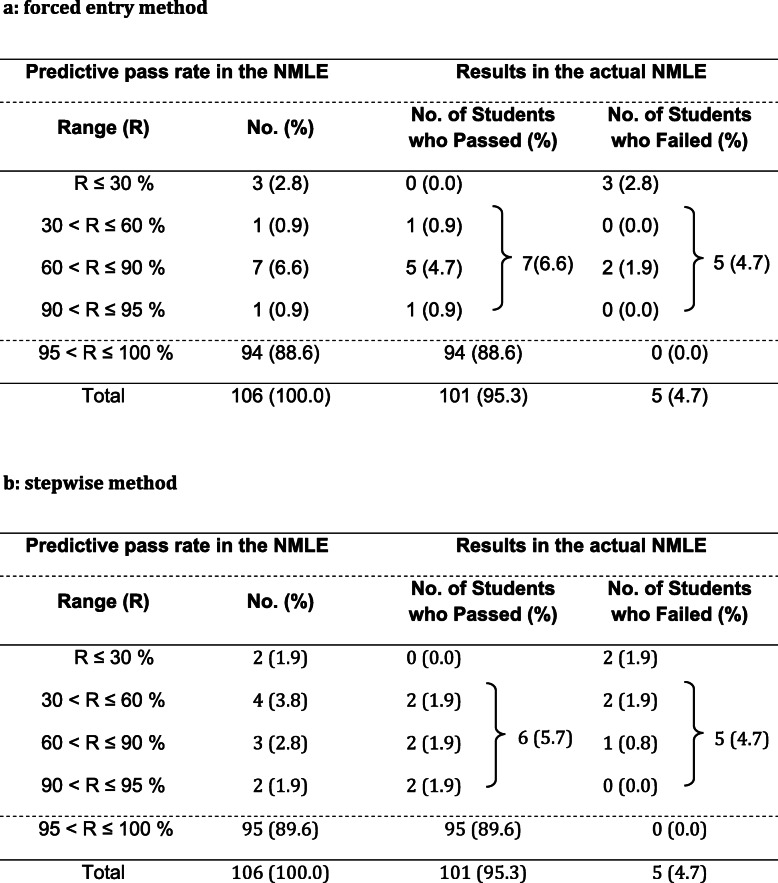
Predictive pass rate and the number of students who failed in the 2018 cohort

*NMLE* National Medical Licensure Examination in Japan. The area of R ≤ 95% means medical students who were judged to need some support prior to graduation and required remediation by the academic affairs committee

## Discussion

We developed a formula for predicting the pass rate in the NMLE. Using this formula, we evaluated a new cohort of students in 2018 and predicted 12 students who had a higher risk of failing the NMLE. After guidance by faculty members, 7 of the 12 students passed the NMLE.

### Predictors for passing the NMLE

We identified four significant internal predictors for passing the NMLE: 1) total score in pre-clinical medical sciences in the third and fourth years, 2) CBT-IRT score in the fourth year, 3) performance in clinical clerkship in the fifth and sixth years, and 4) score in the graduation examination in the sixth year. We also identified two external predictors: age at admission and HS located in surrounding area.

Among them, CBT is a nationwide examination administered by the Common Achievement Tests Organization [26] for medical students in all Japanese medical schools before clinical clerkship using a computer to estimate the student’s knowledge for the clinical clerkship. CBT corresponds to Step 1 of USMLE, and a number of studies on risk factors and outcome for those who failed Step 1 [2–5, 27, 28] and studies investigating Step 1 score as one of the predictors of performance after Step 1 [8–11, 21, 29–32]. The latter may be correlated to our result for CBT. Most studies including the study by Koenig et al. [29] have indicated that a high score in Step 1 is a predictor of success in many fields in the medical profession (i.e., internal medicine, dermatology, ophthalmology, orthopedic surgery, gynecology, and family medicine) [8–11, 30–32], with some opposite results [33, 34]. Casey et al. [21] noted that the medical college aptitude test (MCAT), Step 1 and Step 2, and subsequent clinical performance parameters correlated with NBME scores across all core clinical clerkships. They also emphasized that Step 1 scores identified students at risk of poor performance in NBME subject examinations, facilitating and supporting implementation of remediation before clinical years [21]. Accordingly, it is very reasonable to assume that the score of CBT which is compatible with Step 1 is one of the predictors of passing the NMLE in Japan which is compatible with NBME.

In the present study, additional three other internal predictors for passing the NMLE were also identified: score in pre-clinical medical sciences, performance in clinical clerkship, and graduation examination scores. The NMLE was taken within 3 months after clinical clerkship and graduation examination. The logistic regression analyses in our study showed a negative correlation between the score in pre-clinical medical sciences in the third and fourth academic years and passing the NMLE (Table 2). This result is in conflict with a general thought that the students with higher academic score in preclinical medical science would likely be to pass NMLE. Furthermore, the total scores for pre-clinical medical sciences in the students who failed NMLE in 2012–2017 were actually lower than those who passed NMLE (Table 1). However, when we closely looked into the 36 students who failed, we found that they had older age at admission and better scores in pre-clinical medical sciences but worse performance in the graduation examination. Hence, we hypothesize that older medical students might have insufficient study time because some of them had family or need to work part-time, diminished ability to memorize, or burnout due to longer years of schooling and/or working since they graduated high schools. Further studies are needed to confirm this hypnosis. The four significant internal predictors of passing the NMLE shown in this study can be used to predict those who may fail the NMLE.

Moreover, significant external predictors of passing the NMLE were age at admission and HS located in Gifu and Aichi Prefecture. Using linear regression analysis, Kleshinski et al. [27] identified predictors of performance on Step 1 and Step 2 as follows: science GPA, biologic science section of MCAT, college selectivity, race, and age. Furthermore, McDougle et al. [3] indicated that the relative risk of first-attempt Step 1 failure for medical school graduates was 3.6 for matriculants aged > 22 years (95%CI: 2.0–6.6, *p* < 0.0001). Consequently, older medical students have a higher risk of failing Step 1, Step 2, and the NMLE. It is unclear why medical students who belonged to a neighborhood HS have better chance of passing the NMLE, and we found no such study on the relationship between NMLE and the location of HS or hometown. Given previous study on academic performance [16, 18], students from the neighborhood city/town might be able to receive various kinds of supports from their families physically, economically, and psychologically. Further investigations are required.

### Predicting NMLE with data in lower grade

Several studies have predicted the performance of medical students in Step 1 and primarily focused on first-time test takers [4, 27, 31, 35, 36]. Determining the characteristics of a student who will fail Step 1 is challenging [4] because it is difficult to create models that predict the failure of first-time test takers given the low number of students who fail in most schools [4, 28]. Keeping this in mind, Coumarbatch et al. attempted to create models to predict those who will fail among first-time test takers using logistic regression analysis in 256 students from the graduating class of 2008 at Wayne University [4]. They found that the year-2 standard score and MCAT biological science score were significant predictors of failing and concluded that using internal and external predictors, identifying students at risk of failing Step 1 is possible [4]. Moreover, they described at-risk groups and current educational intervention strategies. In the current study, the year-2 standard score and MCAT score might correspond to the total score in pre-clinical medical science and the NCTUA (Tables **1** and **2**), however, there is a difference of the competencies required and the level of difficulty between MCAT and NCTUA, so it would be reasonable that our results using logistic regression analysis were not consistent with theirs [4]. More recently, Baars et al. developed a model for the early and reliable prediction of students who fail to pass the first year in the undergraduate medical curriculum within 2 years after starting [1]. However, we cannot directly compare our results and theirs. In the GUSM, the students who failed the NMLE did not have better or worse scores in liberal arts and basic science during their first year in medical school (Tables **1** and **2**).

Thus, in the current study, we determined the PPR using several information which can be obtained easily during medical schools, and predicted students who have higher risk to fail NMLE using the PPR for the first time.

### The pass rate in NMLE 2018 after support based on the PPR prediction

Before the current study, faculties noticed that some young students with poor performance in the mock examination (ME) may pass the actual NMLE, while the older students with good performance in the ME sometimes failed NMLE, but the reason was unclear. For a new cohort in 2018, we chose students who had lower PPRs in the NMLE (95% or less), indicating a strong likelihood to fail the NMLE, to confirm the validity of the formula (Table 3). The PPR predicted all five students who would fail. Therefore, this result showed that risk analysis from data such as the PPR can enable effective support from multiple points of view, such as the use of MEs. Further prospective studies are needed in other cultural areas, although we need to confirm the validity of the PPR.

### Limitations

First, we cannot directly compare the present and previous studies because of differences in independent variables. Second, our results may be influenced by some differences in the selection of medical students and the medical education system between Japan and other countries. Third, it may be unclear whether our results can be applied to other Japanese medical schools because there was no report similar to our study and the study period was only 1 year. Therefore, we expect to applicate and verify the knowledge in other Japanese medical schools. Fourth, because Gifu University Graduate School of Medicine is a public education institution, we had no choice but to intervene a group of students with a higher risk to fail NMLE once the risks were identified. As a result, the intervention has made it an incomplete experimental model.

## Conclusions

This is the first study that demonstrated six significant predictors for passing the NMLE and the possibility of decreasing the number of students who fail the NMLE prospectively using the PPR. To confirm these results, further studies are needed because there is no similar trial.

## Supplementary Information

**Additional file 1.**

## Data Availability

Our data are not on a data repository because scores and data of students are highly confidential. The datasets used and/or analyzed during the current study are available from the corresponding author on reasonable request. Only coded data may be shared.

## Abbreviations

AUC: Area Under the Curve
CBT-IRT: Computer-Based Testing with Item Response Theory
GPA: Grade Point Average
GUSM: Gifu University School of Medicine
HS: High School
MCAT: Medical College Aptitude Test
ME: Mock Examination
NBME: National Board of Medical Examiners
NCTUA: National Center Test for University Admissions
NMLE: National Medical Licensure Examination
OECD: Organization for Economic Co-operation and Development
OR: Odds Ratio
PPR: Predictive Pass Rate
Pre-CC OSCE: Pre-Clinical Clerkship Objective Structured Clinical Examination
TOEFL: Test of English as a Foreign Language
USMLE: United States Medical Licensing Examination

## References

[1] Baars G, Stijnen T, Splinter T (2017). A model to predict student failure in the first year of the undergraduate medical curriculum. Health Profess Educ.

[2] Burns ER, Garrett J (2015). Student failures on first-year medical basic science courses and the USMLE step 1: a retrospective study over a 20-year period. Anat Sci Educ.

[3] McDougle L, Mavis BE, Jeffe DB (2013). Academic and professional career outcomes of medical school graduates who failed USMLE step 1 on the first attempt. Adv Health Sci Educ Theory Pract.

[4] Coumarbatch J, Robinson L, Thomas R (2010). Strategies for identifying students at risk for USMLE step 1 failure. Fam Med.

[5] Biskobing DM, Lawson SR, Messmer JM (2006). Study of selected outcomes of medical students who fail USMLE step 1. Med Educ Online.

[6] Kies SM, Freund GG (2005). Medical students who decompress during the M-1 year outperform those who fail and repeat it: a study of M-1 students at the University of Illinois College of Medicine at Urbana-Champaign 1988–2000. BMC Med Educ.

[7] Physician Licensing Service (2020). Medical License Requirements by State.

[8] Fening K, Vander Horst A, Zirwas M (2011). Correlation of USMLE step 1 scores with performance on dermatology in-training examinations. J Am Acad Dermatol.

[9] Swanson DB, Sawhill A, Holtzman KZ (2009). Relationship between performance on part I of the American Board of Orthopaedic Surgery Certifying Examination and Scores on USMLE steps 1 and 2. Acad Med.

[10] McCaskill QE, Kirk JJ, Barata DM (2007). USMLE step 1 scores as a significant predictor of future board passage in pediatrics. Ambul Pediatr.

[11] Myles TD, Henderson RC (2002). Medical licensure examination scores: relationship to obstetrics and gynecology examination scores. Obstet Gynecol.

[12] Case SM, Swanson DB (1993). Validity of NBME part I and part II scores for selection of residents in orthopedic surgery, dermatology, and preventive medicine. Acad Med.

[13] USMLE (2017). Performance data.

[14] Japanese Ministry of Health, Labour and Welfare. Performance Dara for the 112th National Medical Licensure Examination in Japan (in Japanese). www.mhlw.go.jp/file/05-Shingikai-10803000-Iseikyoku-Ijika/0000197914.pdf Accessed June 2, 2020.

[15] Ferguson E, James D, Madeley L (2002). Factors associated with success in medical school: systematic review of the literature. BMJ..

[16] Nawa N, Numasawa M, Nakagawa M, Sunaga M, Fujiwara T, Tanaka Y, Kinoshita A (2020). Associations between demographic factors and the academic trajectories of medical students in Japan. PLoS One.

[17] Cleland JA, Milne A, Sinclair H, Lee AJ (2008). Cohort study on predicting grades: is performance on early MBChB assessments predictive of later undergraduate grades?. Med Educ.

[18] Malau-Aduli BS, O’Connor T, Ray RA, van der Kruk Y, Bellingan M, Teague PA (2017). Risk factors associated with academic difficulty in an Australian regionally located medical school. BMC Med Educ.

[19] Kumwenda B, Cleland JA, Walker K, Lee AJ, Greatrix R (2017). The relationship between school type and academic performance at medical school: a national, multi-cohort study. BMJ Open.

[20] O’Neill LD, Wallstedt B, Eika B, Hartvigsen J (2011). Factors associated with dropout in medical education: a literature review. Med Educ.

[21] Casey PM, Palmer BA, Thompson GB (2016). Predictors of medical school clerkship performance: a multispecialty longitudinal analysis of standardized examination scores and clinical assessments. BMC Med Educ.

[22] Suzuki Y, Gibbs T, Fujisaki K (2008). Medical education in Japan: a challenge to the healthcare system. Med Teach.

[23] Imafuku R, Saiki T, Suzuki Y (2016). Developing undergraduate research in Japanese medical education. Council Undergrad Res Q.

[24] Organization for Economic Co-operation and Development OECD Data: Doctors. https://data.oecd.org/healthres/doctors.htm Accessed June 2, 2020.

[25] Organization for Economic Co-operation and Development OECD Data: Medical graduates. https://data.oecd.org/healthres/medical-graduates.htm Accessed June 2, 2020.

[26] Yamada R. Measuring quality of undergraduate education in Japan: comparative perspective in a knowledge based society. Elsevier. 2014:76–8.

[27] Kleshinski J, Khuder SA, Shapiro JI (2009). Impact of preadmission variables on USMLE step 1 and step 2 performance. Adv Health Sci Educ Theory Pract.

[28] Zhao X, Oppler S, Dunleavy D (2010). Validity of four approaches of using repeaters MCAT scores in medical school admissions to predict USMLE step 1 total scores. Acad Med.

[29] Koenig JA, Sireci SG, Wiley A (1998). Evaluating the predictive validity of MCAT scores across diverse applicant groups. Acad Med.

[30] Myles T, Galvez-Myles R (2003). USMLE step 1 and 2 scores correlate with family medicine clinical and examination scores. Fam Med.

[31] Crawford CH, Nyland J, Roberts CS, Johnson JR (2010). Relationship among United States medical licensing step I, orthopedic in-training, subjective clinical performance evaluations, and American Board of Orthopedic Surgery Examination Scores: a 12-year review of an orthopedic surgery residency program. J Surg Edu.

[32] Hemann BA, Durning SJ, Kelly WF, Dong T, Pangaro LN (2015). Referral for competency committee review for poor performance on the internal medicine clerkship is associated with poor performance in internship. Mil Med.

[33] Herndon JH, Allan BJ, Dyer G, Jawa A, Zurakowski D (2009). Predictors of success on the American Board of Orthopaedic Surgery Examination. Clin Orthop Relat Res.

[34] Johnson GA, Bloom JN, Szczotka-Flynn L, Zauner D, Tomsak RL (2010). A comparative study of resident performance on standardized training examinations and the American board of ophthalmology written examination. Ophthalmology.

[35] Julian ER (2005). Validity of the medical college admission test for predicting medical school performance. Acad Med.

[36] Donnon T, Paolucci EO, Violato C (2007). The predictive validity of the MCAT for medical school performance and medical board licensing examinations: a meta-analysis of the published research. Acad Med.

