# Supplementary material for: Identifying and supporting students at risk of failing the National Medical Licensure Examination in Japan using a predictive pass rate
Source: BMC Med Educ. 2020 Nov 10;20:419. doi: 10.1186/s12909-020-02350-8 (PMC7654142; doi:10.1186/s12909-020-02350-8)
Supplement: Supplementary file 1 — Additional file 1. [file 12909_2020_2350_MOESM1_ESM.docx]

**Additional file 1: Logistic regression formula of PPR in the NMLE**

Forced entry method: log{p/(1 − p)} = 7.75849－0.13301 × age at admission + 1.00772 × female + 1.60261 × neighborhood − 0.77012 × type of HS (public) − 0.03346 × level of HS + 0.26591 × HS GPA + 0.03069 × NCTUA score − 0.02104 × score in liberal arts + 0.00456 × TOEFL score + 0.03728 × score in basic sciences in the first year + 0.09396 × score in basic biomedical sciences in the second year − 0.35949 × score in pre-clinical medical sciences from the third to fourth years + 0.16101 × CBT-IRT score − 0.05099 × Pre-CC OSCE score + 1.42424 × performance in clinical clerkship from the fifth to sixth years + 1.72797 × achievement in the graduation examination + 0.68164 × with holdover.

Stepwise method: log{p/(1 − p)} = 11.65386－0.15522 × age at admission + 1.57629 × neighborhood − 0.25435 × score in pre-clinical medical sciences from the third to fourth years + 0.16662 × CBT-IRT score + 1.4537 × performance in clinical clerkship from the fifth to sixth years + 1.70081 × achievement in the graduation examination.
